# Supplementary material for: Thermoneutral housing has limited effects on social isolation-induced bone loss in male C57BL/6J mice
Source: JBMR Plus. 2025 May 11;9(7):ziaf088. doi: 10.1093/jbmrpl/ziaf088 (PMC12202045; doi:10.1093/jbmrpl/ziaf088)
Supplement: Supplementary_Materials_2025_04_18_ziaf088 [file supplementary_materials_2025_04_18_ziaf088.pdf]

Supplementary Materials

Supplementary Table S1. Primer sequences used for qPCR.

Gene	Abbreviation	Forward Primer Sequence	Reverse Primer Sequence	Manufacturer
Beta actin	*Actb*	NA	NA	Qiagen (#PPM02945B)
Beta 1 adrenergic receptor	*Adrb1*	NA	NA	Qiagen (#PPM05035A)
Beta 2 adrenergic receptor	*Adrb2*	NA	NA	Qiagen (#PPM04265C)
Colony stimulating factor 1 receptor	*Csf1r*	NA	NA	Qiagen (#PPM03625F)
Type II deiodinase	*Dio2*	5'-CAG TGT GGT GCA CGT CTC CAA TC-3'	5'-TGA ACC AAA GTT GAC CAC CAG-3'	IDT[1]
Hydroxysteroid 11-Beta Dehydrogenase 1	*Hsd11b1*	5'-TCA TGG GCG GAC TGG ACA T-3'	5'-AGC AAT CAT AGG CTG GGT CAT-3'	PrimerBank (#113680740c2)
Nuclear Receptor Subfamily 3 Group C Member 1 (Glucocorticoid receptor)	*Nr3c1*	5'-AGC TCC CCC TGG TAG AGA-3'	5'-GGT GAA GAC GCA GAA ACC TTG-3'	PrimerBank (#6680103a1)
Pyruvate dehydrogenase lipoamide kinase isozyme 4	*Pdk4*	5'-AAA GTG GGT CTG TGG CAT TG-3'	5'-AAG GTT TGT ACT CGT GTT TGT G-3'	Primer Design
Peroxisome proliferative activated receptor, gamma, coactivator 1 beta	*Ppargc1a*	5'-TGA TGT GAA TGA CTT GGA TAC AGA CA-3'	5'-GCT CAT TGT TGT ACT GGT TGG ATA TG-3'	IDT[1]
NF-κB subunit RelA/p65	*Rela*	NA	NA	Qiagen (#PPM04224F)
Runt-related transcription factor 2	*Runx2*	5'-GAC AGA AGC TTG ATG ACT CTA AAC C-3'	5'-TCT GTA ATC TGA CTC TGT CCT TGT G-3'	IDT[2]
SH3 and PX domains 2A	*Sh3pxd2a*	NA	NA	Qiagen (#PPM38695A)
Osteoprotegerin (OPG)	*Tnfrsf11b*	NA	NA	Qiagen (#PPM03404F)
Receptor activator of nuclear factor kappa-B ligand (RANKL)	*Tnfsf11*	NA	NA	Qiagen (#PPM03047F)
Uncoupling protein 1	*Ucp1*	5'-ACT GCC ACA CCT CCA GTC ATT-3'	5'-CTT TGC CTC ACT CAG GAT TGG-3	IDT[1]

1. Cooper MP, Uldry M, Kajimura S, Arany Z, Spiegelman BM. Modulation of PGC-1 coactivator pathways in brown fat differentiation through LRP130. J. Biol. Chem. 2008
2. Ontiveros C, Irwin R, Wiseman RW, McCabe LR. Hypoxia suppresses runx2 independent of modeled microgravity. J Cell Physiol [Internet]. 2004;200(2):169–76. Available from: http://www.ncbi.nlm.nih.gov/pubmed/15174088

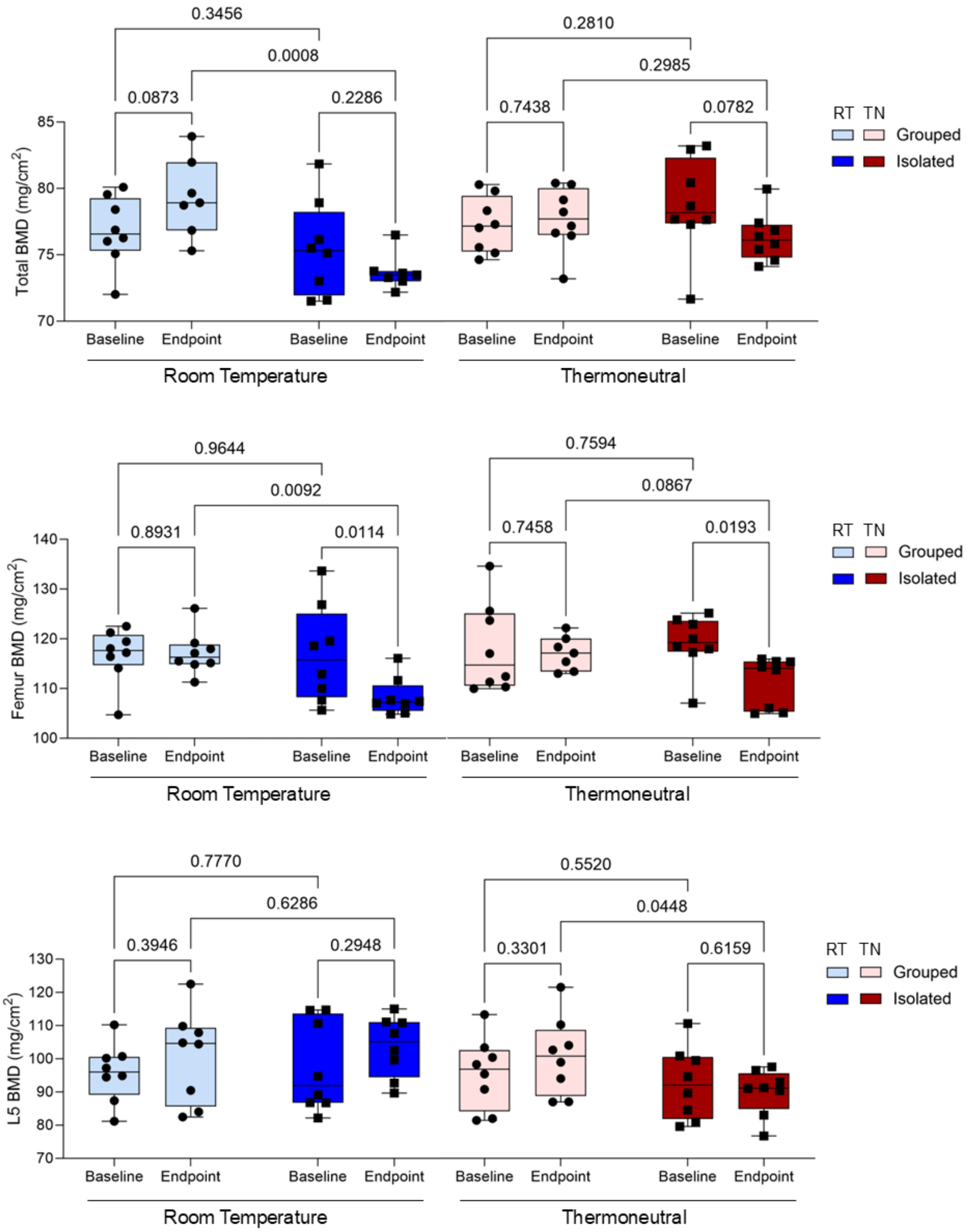

Supplemental Figure S1. **Social isolation reduced femoral BMD relative to baseline**. Bone parameters were measured using dual-energy x-ray absorptiometry (DXA) at baseline (16 weeks of age) and endpoint (after 4 weeks of treatment). p-values from room temperature and thermoneutral 2-way ANOVA pairwise comparisons shown on graphs. N=7-8/group.

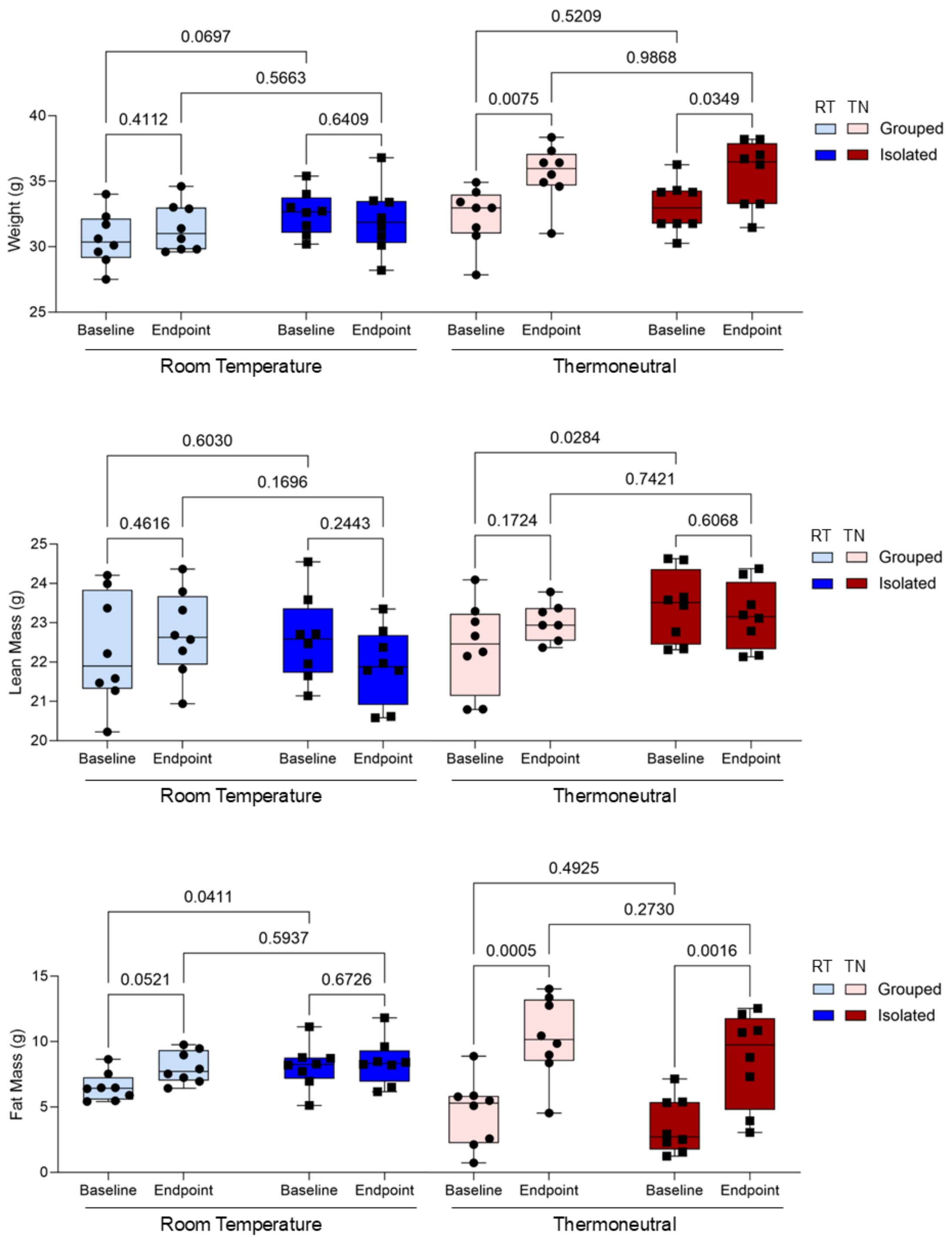

Supplemental Figure S2. **Thermoneutral housing increased weight and fat mass relative to baseline**. Lean and fat mass were measured using dual-energy x-ray absorptiometry (DXA) and weight was measured at baseline (16 weeks of age) and endpoint (after 4 weeks of treatment). p-values from room temperature and thermoneutral 2-way ANOVA pairwise comparisons shown on graphs. N=7-8/group.

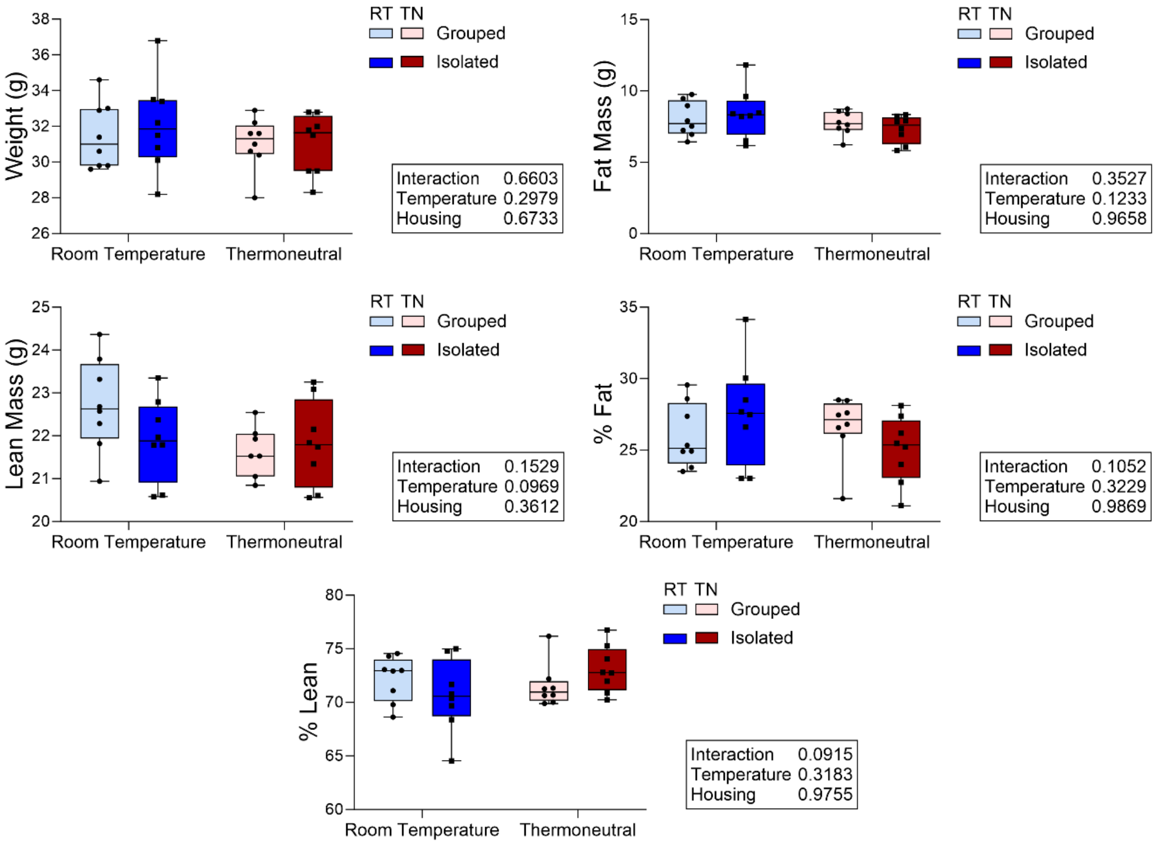

Supplemental Figure S3. **Housing and temperature did not significantly alter endpoint body parameters**. At the end of 4 weeks of treatment, body parameters were measured using dual-energy x-ray absorptiometry (DXA). N=7-8/group.
